# Secondary Syphilis Presenting as Fulminant Hepatic Failure: A Case Report and Literature Review

**DOI:** 10.7759/cureus.78887

**Published:** 2025-02-12

**Authors:** Ignacio Gondolesi, Carlos A Vigliano, Andrés Fraile, Valeria I Descalzi, Luis C Perez Illidge

**Affiliations:** 1 Hepatology, Universidad Favaloro, Buenos Aires, ARG; 2 Pathology, Fundación Favaloro, Buenos Aires, ARG; 3 Transplant, Fundación Favaloro, Buenos Aires, ARG; 4 Hepatology, Fundación Favaloro, Buenos Aires, ARG; 5 Critical Care Medicine, Fundación Favaloro, Buenos Aires, ARG

**Keywords:** fulminant hepatic failure, hepatitis, liver transplant, secondary syphilis, sexually transmitted disease, syphilis, transplant

## Abstract

Syphilis has afflicted humanity for centuries, and despite advances in prevention and treatment, its prevalence continues to rise. Certain forms of the disease remain underreported, contributing to an incomplete understanding of their presentations. This report details the case of a 20-year-old female who developed fulminant hepatic failure (FHF) associated with active secondary syphilis. Despite receiving penicillin treatment, she required a liver transplant. To raise awareness in the medical community, the authors also review similar cases from the literature. Notably, only two other reports of FHF linked to secondary syphilis in adults exist, highlighting the limited experience with this presentation. All three cases shared similar clinical features, and each patient met the criteria for placement on the liver transplant waiting list. Given the rising incidence and diverse manifestations of syphilis, *Treponema pallidum* infection should be considered a potential cause of acute liver failure in sexually active patients. The similarities among these cases suggest a recognizable pattern of signs and symptoms, underscoring the importance of early referral to a transplant center.

## Introduction

Syphilis is a chronic systemic infection caused by the *Treponema pallidum *spirochete. Despite extensive public health efforts to control its spread, syphilis transmission has increased globally since the 2000s [1,2]. In Argentina, the annual incidence of syphilis tripled between 2010 and 2019 compared to the early 2000s, with the latest national report in 2023 identifying females aged 20 to 24 as the most affected group [3,4]. A similar trend has been observed worldwide; for instance, in the United States, reported cases to the CDC rose by 81% between 2014 and 2018 [5].

The spirochete spreads through sexual contact, blood transfusion, or transplacental transmission. Syphilis progresses through distinct stages: primary (painless chancre), secondary - often called “the great imitator” - (non-pruritic rash primarily affecting the palms and soles, fever, lymphadenopathy, and mucosal lesions), latent (asymptomatic), and tertiary, which may involve ocular, cardiovascular, gummatous syphilis, or neurosyphilis [5,6].

Liver involvement can occur at any stage, manifesting as syphilitic hepatitis or part of gummatous disease, both of which are well documented in the literature [5,7]. However, fulminant hepatic failure (FHF) due to syphilis remains exceedingly rare.

This study aims to report the case of a young female adult who developed FHF while experiencing secondary syphilis and subsequently required a liver transplant. Additionally, a literature review is conducted to explore FHF as a rare manifestation of secondary syphilis, contributing to a better understanding of its incidence and clinical significance.

## Case presentation

Methods

To conduct this case report, the patient’s electronic clinical records were reviewed, focusing on an uncommon complication of secondary syphilis. Additionally, a comprehensive literature search was performed using PubMed, SciELO, and LILACS databases, as well as citation searching, to identify cases of adults who developed FHF in the context of secondary syphilis.

The search utilized the following Medical Subject Headings (MeSH) terms: Fulminant Hepatic Failure and Syphilis. As of February 2025, 15 relevant papers were identified. Only studies describing adults with new-onset FHF during secondary syphilis were included in the analysis. Articles reporting liver involvement with a severity level different from FHF, or those lacking clinical, laboratory, or anatomopathological data, were excluded.

Results

Case Report

A 20-year-old female with no significant medical history was referred to our transplant center due to the new onset of jaundice, which began seven days before admission, followed by vomiting. Coagulopathy was noted the day before her referral.

**Figure 1:**
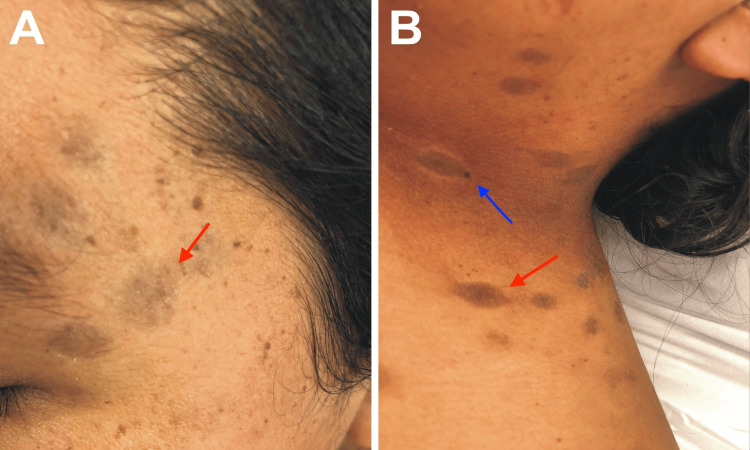
Skin findings on admission Hyperpigmented macular lesions (red arrows) around the eyes (A) and on the side of the neck (B), some of which are surrounded by a hypopigmented halo (blue arrow).

On physical examination, non-pruritic, non-desquamative macules were present on the palms and soles, along with hyperpigmented macular lesions in the perioral region, on the bridge of the nose, along the hairline, and on the back of the neck (Figure 1). Additionally, hyperpigmented macules with areas of desquamation were observed on the abdomen, trunk, and elbow folds. The patient had noticed these lesions along with the onset of jaundice.

Upon admission to the transplant center, blood tests were performed. The most notable findings included hypoalbuminemia, hyperbilirubinemia, elevated liver enzymes, and coagulopathy (Table 1).

**Table 1:** Laboratory tests on admission ALP: alkaline phosphatase; ALT: alanine aminotransferase; APTT: activated partial thromboplastin time; AST: aspartate aminotransferase; PT-INR: prothrombin time-international normalized ratio

Parameter	Value	Reference range
Coagulation tests		
PT-INR	2.36	0.9-1.15
APTT (seconds)	76.8	25.0-37.0
Liver function tests		
AST (U/liter)	1132	5-32
ALT (U/liter)	898	10-35
ALP (U/liter)	115	40-150
Total bilirubin (mg/dl)	19.5	0.2-1.0
Direct bilirubin (mg/dl)	15.5	0.1-0.5
Serum chemistry		
Total protein (g/dl)	5.7	6.3-8.3
Albumin (g/dl)	2.58	3.5-5.2
Lactate dehydrogenase (U/liter)	331	45-200

According to the transfer medical records, the patient had a positive venereal disease research laboratory (VDRL) test. Consequently, the study plan was expanded to include a repeat VDRL test and a treponemal test, both of which returned positive. Intravenous sodium penicillin G was initiated and continued for 14 days.

On the first day of admission, the patient developed signs of hepatic encephalopathy, including bradyphrenia and asterixis. Concurrently, follow-up blood tests revealed worsening coagulopathy and increased ammonia levels, indicating severe liver damage and progression to FHF.

**Figure 2:**
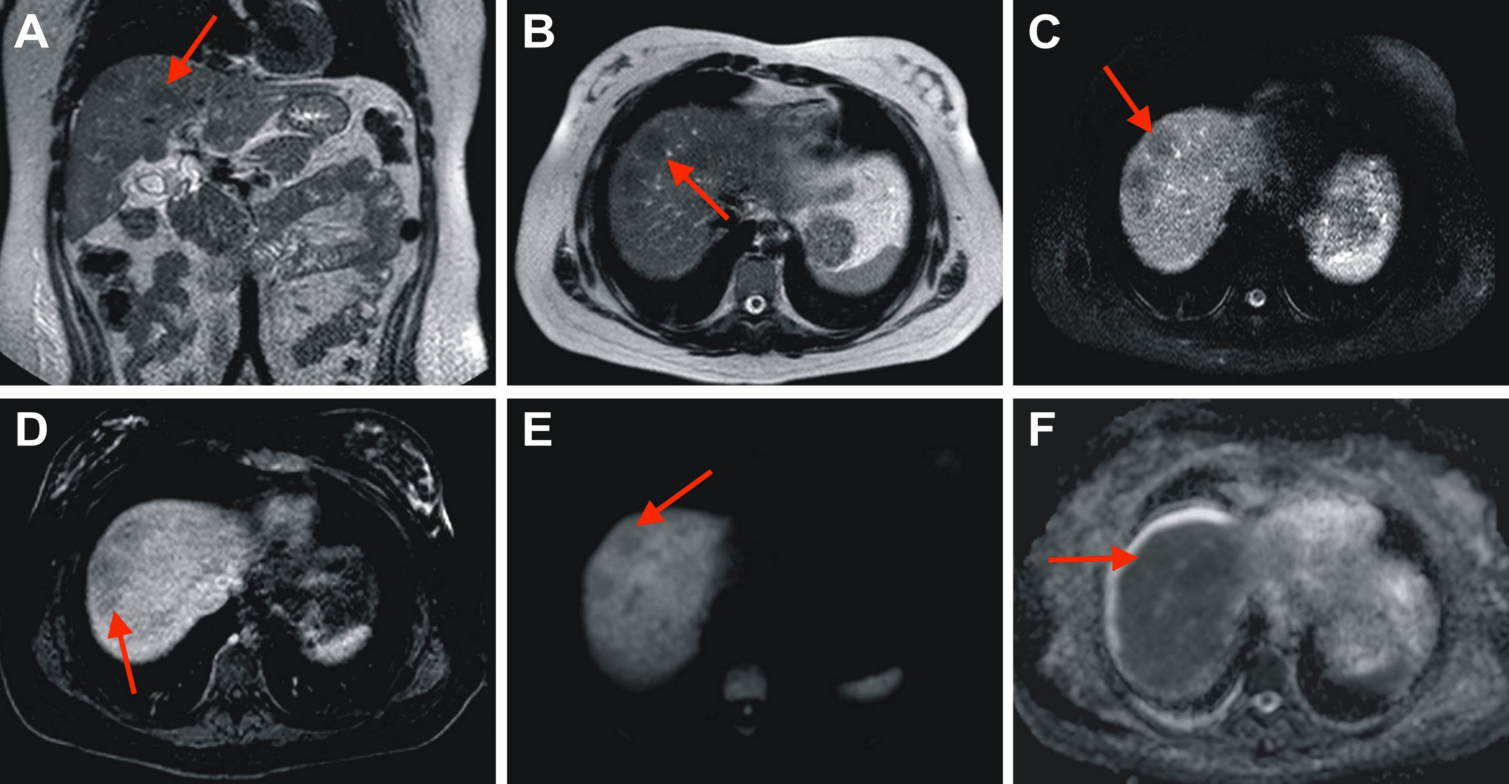
Abdominal MRI obtained during the patient’s initial evaluation Abdominal MRI with gadolinium contrast. (A) Coronal T2. (B) Axial T2. (C) Axial SPAIR. (D) Axial T1-weighted image with gadolinium (portal phase). (E) Axial diffusion-weighted imaging. (F) Axial apparent diffusion coefficient map. The liver is of preserved size and has regular contours, but it exhibits heterogeneous signal intensity due to multiple focal, nodular lesions (arrows). These lesions appear hypointense on T2/SPAIR sequences (A, B, C), isointense on T1, with no enhancement following intravenous gadolinium administration (D), and show no diffusion restriction (E, F).

Multiple diagnostic tests were conducted, including an autoantibody panel, viral serologies for HAV, HCV, HBV, HEV, and HIV, urine toxicology screening, and metabolic studies (serum and 24-hour urine copper levels, ceruloplasmin, and alpha-1 antitrypsin phenotype), ruling out the most common causes of acute liver failure. Additionally, an abdominal MRI was performed to assess structural abnormalities of the liver (Figure 2).

Given the progression to FHF and the fulfillment of three minor criteria according to King’s College criteria - (1) jaundice persisting for more than seven days before the onset of encephalopathy; (2) total bilirubin levels exceeding 17.5 mg/dL; and (3) prothrombin time greater than 50 seconds (international normalized ratio > 3.5) [8] - the patient was listed for an emergency liver transplant.

Supportive treatment was initiated but failed to improve clinical parameters, and liver synthetic function continued to decline, as evidenced by worsening laboratory results. Forty-eight hours after being placed on the transplant waiting list, a compatible liver donor was identified, and an orthotopic liver transplant was performed. The donor’s serology was positive for toxoplasmosis and VDRL.

To complete the diagnostic workup, samples from the explanted liver were obtained during transplant surgery and analyzed by the pathology department. Routine staining techniques, including PAS-D, Perls’, Masson’s trichrome, and reticulin, were performed to detect spirochetes, all of which were negative. Additionally, Warthin-Starry staining and Treponema immunohistochemistry were conducted, but no spirochetes were identified. Immunohistochemical techniques for viral pathogens (adenovirus, CMV, HBV, HSV-1, and HSV-2) were also applied to histological sections up to 5-µm thick, all of which were negative.

**Figure 3:**
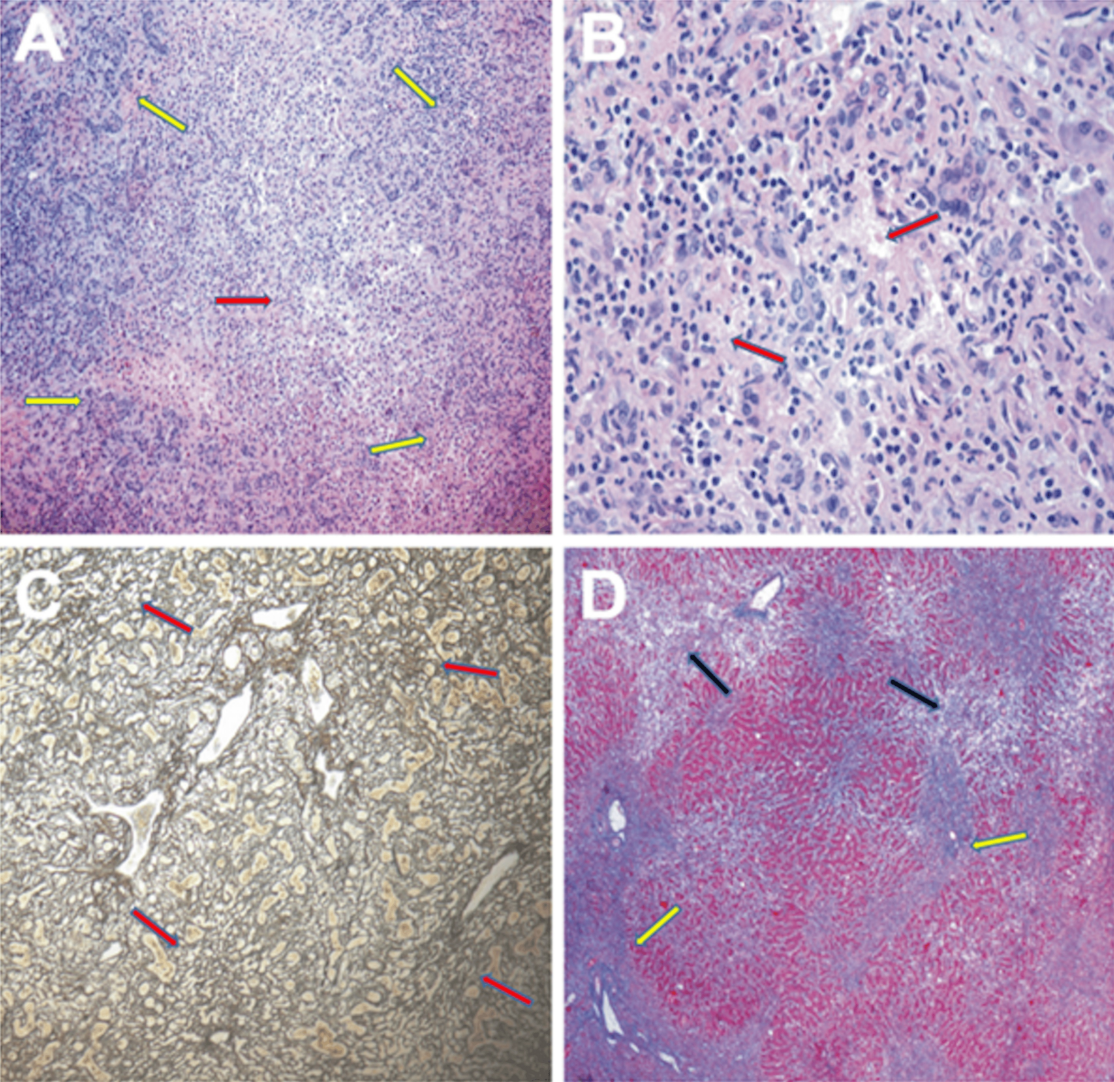
Histological findings from the explanted liver samples (A) Liver parenchyma showing necrosis and hepatocyte loss, predominantly in zone 3 (red arrow), with replacement by pseudo-acinar type regeneration and reparative mesenchymal tissue (yellow arrows) (H&E, original magnification ×100). (B) The explanted liver exhibits extensive hepatic parenchymal destruction, patchy necrosis, lymphocytic infiltrates, and bile duct proliferation with accompanying neutrophils (red arrows), without granuloma formation or viral cytopathic effects (HE, original magnification ×200). (C) Liver parenchyma demonstrating histoarchitectural disorganization with areas of reticular framework collapse (arrows) (reticulin stain, original magnification ×200). (D) Portal fibrous expansion with porto-portal and porto-central fibroinflammatory septa (yellow arrows), interspersed with areas of necrosis and liver parenchymal collapse (black arrows) (Masson’s trichrome, original magnification ×200).

Histological findings were consistent with FHF, characterized by sub-massive hepatocyte necrosis, marked hepatocellular cholestasis, and an inflammatory infiltrate predominantly composed of lymphocytes with periportal and lobular distribution (Figure 3).

Liver enzyme levels gradually declined, reaching normal values by six days post-transplant. The patient received intravenous corticosteroids during engraftment and throughout the first post-transplant week, along with extended-release tacrolimus starting on postoperative day 1 as maintenance immunosuppression.

During hospitalization, the cutaneous lesions observed at admission showed progressive improvement, with a marked resolution noted 12 days after initiating sodium penicillin G. The patient was discharged on post-transplant day 17.

Literature Review

**Figure 4:**
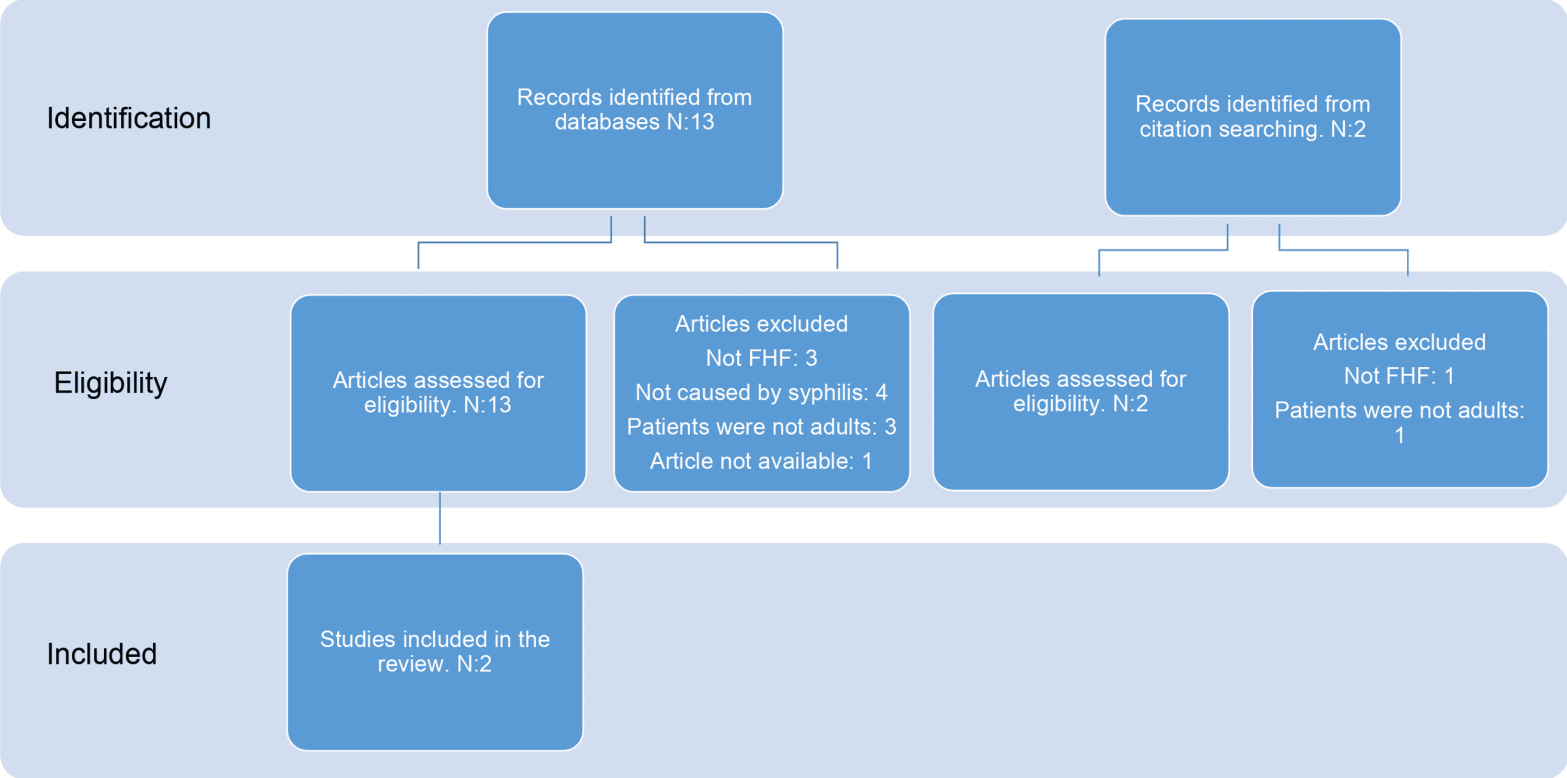
Flow diagram showing the article selection process

Of the 15 papers identified in our search, only six reported cases of FHF associated with syphilis. Four of these involved neonates with congenital syphilis - a population in which hepatic involvement occurs in up to 20% of cases [9-12] - and are beyond the scope of this manuscript. The remaining two cases represent, to the best of our knowledge, the only documented instances of FHF in adults with active syphilis. One was reported by Lo et al. in 2007 [13], and the other by Affonso da Costa et al. in 2018 [14]. The selection process is illustrated in Figure 4.

These two cases presented similarly to ours. Both patients were 18-year-old females with no significant medical history who developed nausea, vomiting, and jaundice associated with macular skin lesions of varying distribution. Additionally, both cases progressed to coagulopathy and hepatic encephalopathy, though the time between jaundice onset and encephalopathy development differed significantly.

Liver function tests in all three cases followed a similar pattern, with markedly elevated liver transaminases compared to alkaline phosphatase (ALP). Likewise, liver histopathology was consistent across the cases, showing massive or sub-massive hepatic necrosis with an associated inflammatory infiltrate. In all instances, Warthin-Starry staining for spirochetes was negative. Two cases exhibited patchy necrosis, while the third showed ductal proliferation.

Ultimately, all three patients required emergency liver transplantation. One died while on the waiting list, while the other two received a liver graft and completed syphilis treatment with penicillin during the early post-transplant period.

Table 2 summarizes the cases identified based on the established search criteria and compares them with the present case.

**Table 2:** Comparison of FHF cases associated with secondary syphilis as reported in the scientific literature ALP: alkaline phosphatase; ALT: alanine aminotransferase; AST: aspartate aminotransferase; FHF, fulminant hepatic failure

Criteria	Lo et al. (2007) [13]	Affonso da Costa et al. (2018) [14]	Present case
Sex	Female	Female	Female
Jaundice	Present from onset	Present from onset	Present from onset
Digestive symptoms	Nausea, vomiting, and diarrhea	Nausea, vomiting, and epigastric pain	Nausea and vomiting
Skin lesions	Erythematous macules with central clearance	Hyperkeratotic plaques and macules on palms and soles	Hyperpigmented macular lesions on the peri-oral region, bridge of the nose, back of the neck, trunk, palms, and soles
Type of FHF	Subacute	Acute	Acute
Liver function test results	AST and ALT >>> ALP	AST and ALT >>> ALP	AST and ALT >>> ALP
Pathology findings	Patchy necrosis and lymphocytic infiltrates, bile duct proliferation, surrounded by neutrophils	Massive liver necrosis and intense, multifocal lymphoplasmacytic infiltrate	Sub-massive hepatocyte necrosis, marked hepatocellular cholestasis, lymphocytic infiltrate with periportal and lobular distribution
Warthin-Starry technique	Negative for spirochetes	Negative for spirochetes	Negative for spirochetes
Outcome	Successful liver transplant, favorable outcome	Died on the transplant waiting list	Successful liver transplant, favorable outcome

## Discussion

This report describes a case of secondary syphilis with both cutaneous and hepatic involvement in a young woman. Although the initial finding was cutaneous lesions, hepatic involvement dominated the clinical course, ultimately progressing to FHF. This condition is characterized by rapid liver injury, coagulopathy, and encephalopathy, leading to multi-organ failure in patients without a prior history of liver disease. FHF can be classified into three types based on the timing of encephalopathy onset: (1) hyperacute, occurring within the first seven days of symptom onset; (2) acute, developing between eight and 28 days; and (3) subacute, emerging more than 28 days after symptom onset [8]. Several criteria are used to estimate prognosis, with the King’s College criteria being particularly notable. These criteria provide an objective assessment of disease severity to guide decisions regarding liver transplant eligibility [8].

FHF represents the final stage of liver injury from various causes. The most common etiologies include drugs and toxins (e.g., acetaminophen and *Amanita phalloides*), viral infections (most frequently hepatitis A and B), and miscellaneous conditions such as Wilson’s disease and autoimmune hepatitis [8]. Other less common causes have also been documented, including syphilis.

Hepatic involvement in syphilis is rare. The most typical manifestation is an isolated elevation of transaminases, observed in up to 10% of infected patients. Most cases of syphilitic hepatitis present with a disproportionate increase in ALP relative to aminotransferases [7,15]. While clinically significant syphilitic hepatitis can occur at any stage of the disease, it affects approximately 3% of patients with secondary syphilis. However, fulminant hepatitis and progression to cirrhosis are exceedingly rare [7].

Histopathological examination of syphilitic hepatitis is nonspecific, and direct identification of spirochetes in hepatic biopsy is uncommon [7]. Liver involvement in secondary syphilis has been documented in the literature. Malvar et al. [16] conducted a detailed histopathological analysis of 14 cases, identifying a consistent pattern in five: bile duct injury characterized by ductular reaction, portal tract edema, and lobular cholestasis. Less frequently, other histological features have been described, including an acute hepatitis pattern (lobular disarray, mild-to-moderate lobular inflammation, patchy hepatocyte dropout, and occasional necrosis); fibroinflammatory mass lesions (fibroblastic reaction with mixed lymphoplasmacytic and neutrophilic inflammation surrounding a central necrotizing abscess); and an autoimmune hepatitis-like pattern, marked by chronic hepatitis with prominent portal-based plasma cells and interface hepatitis [16]. A significant finding was positive immunohistochemical staining for *T. pallidum *in 10 patients. However, three negative cases involved patients who had received syphilis treatment shortly before biopsy, similar to the patient in this report [16]. Malvar et al.’s study concluded that secondary syphilitic hepatitis can present with diverse histological patterns and that a negative immunohistochemical result does not exclude the diagnosis [16].

In 2004, Mullick et al. [17] proposed diagnostic criteria for syphilitic hepatitis: (1) elevated liver enzyme levels; (2) serological evidence of syphilis; (3) exclusion of other causes of liver involvement; and (4) resolution of liver enzyme abnormalities following appropriate treatment, typically penicillin [6]. The patient in this report met all criteria except the last, as liver injury was irreversible by the time treatment was initiated, necessitating liver transplantation.

A literature review identified two similar cases [13,14]. Both involved young women with secondary syphilis initially presenting with skin findings, who subsequently developed jaundice and gastrointestinal symptoms, progressing to FHF. Laboratory results revealed comparable liver function test abnormalities, with a more significant increase in transaminases compared to ALP - contrasting with the typical pattern of syphilitic hepatitis [8]. Histopathological findings were also similar. In all three cases, liver dysfunction was severe enough to require transplantation [13,14]. This suggests a distinct pattern of presentation for this complication in secondary syphilis.

Notably, despite the rising incidence of syphilis, only three cases of FHF associated with secondary syphilis have been reported. This suggests that the complication is exceedingly rare or that additional cases may have gone undiagnosed or unreported.

## Conclusions

Given the aggressive progression of hepatitis in these cases and the recent rise in syphilis incidence, *T. pallidum* infection should be considered as a potential etiology in cases of hepatitis and FHF of unknown origin. When this complication is suspected, early consultation with a transplant center should be pursued alongside the standard treatment for the infectious disease.
